# Pathophysiology and Potential Therapeutic Candidates for COVID-19: A Poorly Understood Arena

**DOI:** 10.3389/fphar.2020.585888

**Published:** 2020-09-17

**Authors:** Arghadip Samaddar, Malika Grover, Vijaya Lakshmi Nag

**Affiliations:** Department of Microbiology, All India Institute of Medical Sciences, Jodhpur, India

**Keywords:** coronavirus disease 2019, severe acute respiratory syndrome coronavirus 2, antiviral, anti-inflammatory, drug repurposing, pathophysiology, immunotherapy

## Abstract

Coronavirus disease 2019 (COVID-19), an acute onset pneumonia caused by a novel *Betacoronavirus*, severe acute respiratory syndrome coronavirus 2 (SARS-CoV-2), emerged in the Wuhan City of China in December 2019 and evolved into a global pandemic. To date, there are no proven drugs or vaccines against this virus. Hence, the situation demands an urgent need to explore all potential therapeutic strategies that can be made available to prevent the disease progression and improve patient outcomes. In absence of clinically proven treatment guidelines, several repurposed drugs and investigational agents are currently being evaluated in clinical trials for their probable benefits in the treatment of COVID-19. These include antivirals (remdesivir, lopinavir/ritonavir, umifenovir, and favipiravir), interferon, antimalarials (chloroquine/hydroxychloroquine), antiparasitic drugs (ivermectin and nitazoxanide), biologics (monoclonal antibodies and interleukin receptor antagonist), cellular therapies (mesenchymal stem cells and natural killer cells), convalescent plasma, and cytokine adsorber. Though several observational studies have claimed many of these agents to be effective based on their *in vitro* activities and extrapolated evidence from SARS and Middle East respiratory syndrome (MERS) epidemics, the currently available data remains inconclusive because of ill-defined patient selection criteria, small sample size, lack of concurrent controls, and use of intermediary outcomes instead of patient-relevant outcomes. Moreover, there is a need to clearly define the patient populations who warrant therapy and also the timing of initiation of treatment. Understanding the disease pathology responsible for the clinical manifestations of COVID-19 is imperative to identify the potential targets for drug development. This review explains the pathophysiology of COVID-19 and summarizes the potential treatment candidates, which can provide guidance in developing effective therapeutic strategies.

## Introduction

In December 2019, a cluster of cases of unexplained acute pneumonia was reported from the Wuhan City of China’s Hubei Province. As the causative agent could not be identified, these initial cases were classified as “pneumonia of unknown etiology.” Later on, the cause of this illness was attributed to a novel *Betacoronavirus*, which was designated as 2019-novel coronavirus (2019-nCoV) by the World Health Organization (WHO) (Cascella et al., 2020). On January 30, 2020, as per the International Health Regulations (IHR, 2005), the outbreak was declared a Public Health Emergency of International Concern (PHEIC) by the WHO. On February 11, 2020, the disease was renamed as coronavirus disease 2019 (COVID-19), and on the same day, the Coronavirus Study Group (CSG) of the International Committee on Taxonomy of Viruses designated 2019-nCoV as severe acute respiratory syndrome coronavirus 2 (SARS-CoV-2) due to its phylogenetic similarity with severe acute respiratory syndrome coronavirus (SARS-CoV) (Cascella et al., 2020). Considering its potential to evolve into a pandemic, the WHO raised the threat to the epidemic to the “very high” level on February 28, 2020.^1^ With the alarming increase in the number of COVID-19 cases outside China, affecting thousands of people across several countries, the WHO declared COVID-19 a pandemic on March 11, 2020.^2^ As of August 16, 2020, COVID-19 has affected more than 21 million people across 216 countries and territories, with 761,779 deaths.^3^ USA accounts for the maximum number of cases, followed by Brazil, India, and Russia.

Coronaviruses (CoVs) are a group of enveloped viruses with positive sense single-stranded RNA genome and having a crown-like appearance under an electron microscope. They belong to the order *Nidovirales*, family *Coronaviridae*, and subfamily *Orthocoronavirinae*. Based on genetic and antigenic criteria, CoVs are classified into four genera: *Alphacoronavirus* (α-CoV), *Betacoronavirus* (β-CoV), *Deltacoronavirus* (δ-CoV), and *Gammacoronavirus* (γ-CoV). To date, seven CoVs capable of infecting humans (HCoVs) have been identified (Cascella et al., 2020). According to an estimate, 2% of the population are healthy carriers of CoVs and they account for 5 to 10% of acute respiratory infections (Chen et al., 2020). The common HCoVs, HCoV-OC43 and HCoV-HKU1 (β-CoVs) and HCoV-229E and HCoV-NL63 (α-CoVs), cause mild self-limiting respiratory tract infections. Other human CoVs, SARS-CoV, SARS-CoV-2, and Middle East respiratory syndrome coronavirus (MERS-CoV) (β-CoVs), cause epidemics with variable clinical severity featuring respiratory and extrarespiratory manifestations. SARS-CoV and MERS-CoV infections possess pandemic potential and can cause life-threatening disease with mortality rates up to 10% and 35%, respectively (Cascella et al., 2020). The γ-CoVs infect avian species, while δ-CoVs tend to infect both mammals and birds (Zumla et al., 2016; de Wilde et al., 2018). Phylogenetic analysis has placed SARS-CoV-2 under the subgenus *Sarbecovirus* of the genus *Betacoronavirus*. Next generation sequencing data has revealed that the genome of SARS-CoV-2 bears 96.2% sequence homology with a bat coronavirus RaTG13 and shares 79.5% identity with SARS-CoV (Zheng, 2020). Based on phylogenetic and evolutionary analyses, it has been proposed that both Bat-CoV RaTG13 and SARS-COV-2 might have evolved from a common ancestor, and SARS-CoV-2 might have jumped from bats to humans *via* some unknown intermediate hosts (Perrotta et al., 2020).

COVID-19 is an acute respiratory disease with a clinical spectrum ranging from mild and moderate disease (80%) to severe (15%) and critical illness (5%), with an overall case fatality rate (CFR) of 0.5–2.8%.^4^ The severe and critical illness categories (nearly 20% of all infections) are of special concern in elderly population and those with underlying comorbidities, as the severity and CFR are particularly high in these groups (Perrotta et al., 2020). Several risk factors related to disease severity have been outlined by the United states Centers for Disease Control and Prevention (CDC). Advanced age, male sex, and smoking have been reported as independent risk factors for disease progression, severity, and mortality. It was observed that 20% of the patients in Italy over 80 years of age succumbed to the disease (Livingston and Bucher, 2020), and as per CDC reports, 31–70% of the patients above the age of 85 in the United States required hospitalization (Vishnevetsky and Levy, 2020). A weekly surveillance report by the WHO Regional Office for Europe reported that over 95% of all deaths due to COVID-19 were people aged 60 years or above, and more than 50% were people aged 80 years or older.^5^ It has been hypothesized that there occurs an age-related decline in the clearance of inhaled particles in small airways, possibly due to decrease in the number of cilia and ciliated epithelial cells in the airways (Svartengren et al., 2005). An age-dependent increase in nasal-cavity volume coupled with decreased nasal resistance and upper airway size are other contributory factors (Levitzky, 1984). As age advances, there occurs a disruption of the innate and adaptive arms of the immune system with an impairment of both effector memory T cell and competent B cell functions, along with continuous production of inflammatory mediators and cytokines (inflammaging) (Aw et al., 2007). In healthy state, angiotensin converting enzyme 2 (ACE2) catalyzes the conversion of angiotensin 2 to angiotensin 1−7 and thus, maintains a homeostasis between inflammatory and anti-inflammatory pathways. ACE2 levels have been found to decrease in old age causing elevated angiotensin-2, which increases pulmonary vascular permeability and inflammation, thereby worsening lung injury due to COVID-19 in such patients (Dhochak et al., 2020). Moreover, in the elderly, there occurs an age-related decrease in the vital capacity of lungs and perfusion of vital organs, such as, heart, lungs, and kidneys. SARS-CoV-2 causes a much more severe pneumonia in the aged than younger individuals. It has been observed that the incidence of acute respiratory distress syndrome (ARDS) is higher in the elderly and those with heart, liver and kidney ailments (Perrotta et al., 2020; Wang L. et al., 2020). Also, older age is a surrogate for comorbid illnesses, such as, respiratory and cardiovascular disorders, morbid obesity (i.e., body mass index of ≥40), hypertension, diabetes mellitus, and significant renal and hepatic impairment (Preskorn, 2020). All these risk factors have been linked to higher rates of intensive care unit (ICU) admission, greater disease severity, and poor prognosis. It has been observed that 90% of the patients who require hospitalization, ICU admission, or succumb to the disease have one or more comorbid conditions, irrespective of age (Garg et al., 2020). Therefore, in the elderly, immunosenescence and underlying comorbidities are likely to be the major contributory factors for life-threatening respiratory failure and multisystemic involvement associated with COVID-19.

On the contrary, children develop milder symptoms, rarely require hospitalization, and have an overall better prognosis when compared to adults (Ludvigsson, 2020). A systematic review and meta-analysis including 7780 children with COVID-19 from 26 countries reported milder self-limiting symptoms in majority of the cases, with 0.14% being critical and seven deaths. Unlike adults, children rarely progressed to severe disease requiring ICU admission (Hoang et al., 2020). This can be attributed to the immature immune system in pediatric population, cross-protection from related coronaviruses and other RNA viruses to which they get exposed early in life, competitive inhibition of SARS-CoV-2 by other respiratory viruses simultaneously invading the airways and the lungs, trained non-specific immunity due to childhood immunization (e.g., Bacillus Calmette-Guerin vaccine and Mumps Measles Rubella vaccine), good regenerative capacity of lungs in children, absence of immunosenescence and age-related comorbidities, and high ACE2 expression causing increased metabolism of angiotensin 2 (Dhochak et al., 2020).

Despite overwhelming global efforts, COVID-19 remains a poorly understood disease with limited success in the field of drug development. Understanding the disease pathogenesis is crucial for choosing effective drug targets. This review explains the pathophysiology of COVID-19 and summarizes the potential treatment candidates, which can provide guidance in developing efficient therapeutic strategies.

## SARS-CoV-2 Structure and Pathogenesis

SARS-CoV-2 is a positive sense, single-stranded RNA virus belonging to the genus *Betacoronavirus* (subgenus *Sarbecovirus*, subfamily *Orthocoronavirinae*). The genomic mRNA has a 5´-cap and a 3´-poly (A) tail and can act as an mRNA for translation of the viral polyproteins. In addition, both 5´ and 3´ ends of the genomic RNA contain a highly structured untranslated region (UTR) that plays an important role in the regulation of RNA replication and transcription. The SARS-CoV-2 genome contains 14 open reading frames (ORFs), preceded by transcriptional regulatory sequences (TRSs). The two main transcriptional units, ORF1a and ORF1ab, comprise two-thirds of the viral genome and encode two major polyproteins: pp1a (~500 kDa) and pp1ab (~800 kDa), respectively. The synthesis of pp1ab involves programmed ribosomal frame shifting during translation of ORF1a. These polypeptides are cleaved by virally encoded chymotrypsin‐like protease (3CLpro), main protease (Mpro), and papain‐like protease (PLpro) into 16 non-structural proteins (nsp1-nsp16), which assemble to form the replication-transcription complex (RTC) involved in genome transcription and replication (Naqvi et al., 2020; Romano et al., 2020). pp1a is cleaved into 11 non-structural proteins (nsp1–nsp11) and pp1ab into five (nsp12–nsp16). The non-structural proteins play an important role in the pathogenesis of COVID-19. nsp3 and nsp5 encode PLpro and 3CLpro, respectively, which help in peptide cleaving and host innate immune antagonism. nsp12 and nsp15 encode RNA-dependent RNA polymerase (RdRp) and RNA helicase, respectively. Other ORFs at the 3´ end of the viral genome encode four structural proteins: the spike surface glycoprotein (S), membrane (M), envelope (E), and the nucleocapsid (N) proteins, which are the major components of the virus playing a crucial role in structural integrity and pathogenesis (Romano et al., 2020; Wu et al., 2020). The S protein is a homotrimeric transmembrane glycoprotein that determines diversity to coronaviruses and host tropism. It has two functional subunits: S1, responsible for binding to the host ACE2 receptors and S2, for the fusion of the virion and cellular membranes. The M protein helps in transport of nutrients across the cell membrane, bud release, and the formation of viral envelope. The E protein plays a significant role in viral morphogenesis and assembly. The N protein plays an important role in packaging of viral RNA into ribonucleocapsid and also helps in immune evasion by attenuating host immune responses (Astuti, 2020; Naqvi et al., 2020). Besides these structural proteins, the 3´ end also contains eight putative ORFs for accessory proteins: 3a, 3b, p6, 7a, 7b, 8b, 9b, and ORF14. The structural and accessory proteins are translated from a set of nested sub-genomic RNAs (sgRNAs) (Wu et al., 2020).

### SARS-CoV-2 Invasion Into Host Cells

The life cycle of SARS-CoV-2 consists of five steps: attachment, penetration, biosynthesis, maturation, and release. Entry of the virus into the host cells is facilitated by interactions between the S protein and its receptors, ACE2, which are found in various organs such as heart, lungs, kidneys, and gastrointestinal tract. The S protein binds to ACE2 through the receptor binding domain (RBD) region of the S1 subunit, which consists of a core and a receptor binding motif (RBM). RBM specifically recognises human ACE2 as its receptor (Yuki et al., 2020). The S protein/ACE2 interaction (attachment) is the primary determinant to infect a host species and also controls tissue tropism. ACE2 mediates human-to-human transmission, and also acts as a receptor for SARS-CoV and respiratory coronavirus NL63 (Astuti, 2020). Following binding of the virus to the host ACE2 receptors, the S protein undergoes a two-step sequential proteolytic cleavage, one at S1/S2 cleavage site for priming and another at S2ˊ site for activation. The latter acts as a viral fusion peptide that inserts into the membrane, followed by the joining of two heptad repeats in S2 forming a six-helix bundle. The formation of this bundle results in fusion and entry of virus into the host cell (penetration) (Tang et al., 2020). Another receptor, which has found to be of importance in viral invasion, is cluster of differentiation 147 (CD147), also known as extracellular matrix metalloproteinase inducer (EMMPRIN) or Basigin (Wang K. et al., 2020). A characteristic unique to SARS-CoV-2 is the existence of a novel furin cleavage site (PRRARS) at S1/S2, which confers the ability to infect organs and tissues where furin is ubiquitously expressed such as the brain, lung, liver, gastrointestinal tract, and pancreas (Wang Q. et al., 2020). Other proteases that may play a role in virus entry are transmembrane protease serine 2 (TMPRSS2) and cathepsin L. Following internalization, there is uncoating and release of viral ssRNA in the host cell cytoplasm, which then gets attached to the ribosomes and is translated into two large polyproteins, pp1a and pp1ab. These polyproteins are cleaved by virus-encoded proteinases into 16 nsps. Many of these non-structural proteins congregate to form the RTC in double-membrane vesicles (DMVs), which are mainly an assembly of RdRp- and helicase-containing subunits (Astuti, 2020; Chen et al., 2020). Synthesis of genomic RNA follows the translation and assembly of viral replicase complexes. RTC is responsible for RNA replication and transcription of the sgRNAs. The latter serve as mRNAs for the translation of structural and accessory proteins (biosynthesis). Following translation, the S, E, and M proteins are transported to the endoplasmic reticulum where they move along the secretory pathway into the endoplasmic reticulum–Golgi intermediate compartment (ERGIC). In the compartment, the viral genomes are encapsidated by the N protein, which then bud into the membrane resulting in formation of the mature virus (maturation) (Fehr and Perlman, 2015). The M protein regulates most of the protein-protein interactions required for virus assembly. However, virus-like particles (VLPs) can only be formed when M protein is co-expressed with E protein, suggesting the role of these two proteins for production of viral envelope. Following assembly, the virions are transported to the cell surface in vesicles and released by exocytosis (release) (Fehr and Perlman, 2015; Astuti, 2020).

### Host Immune Response to SARS-CoV-2

**Figure 1:**
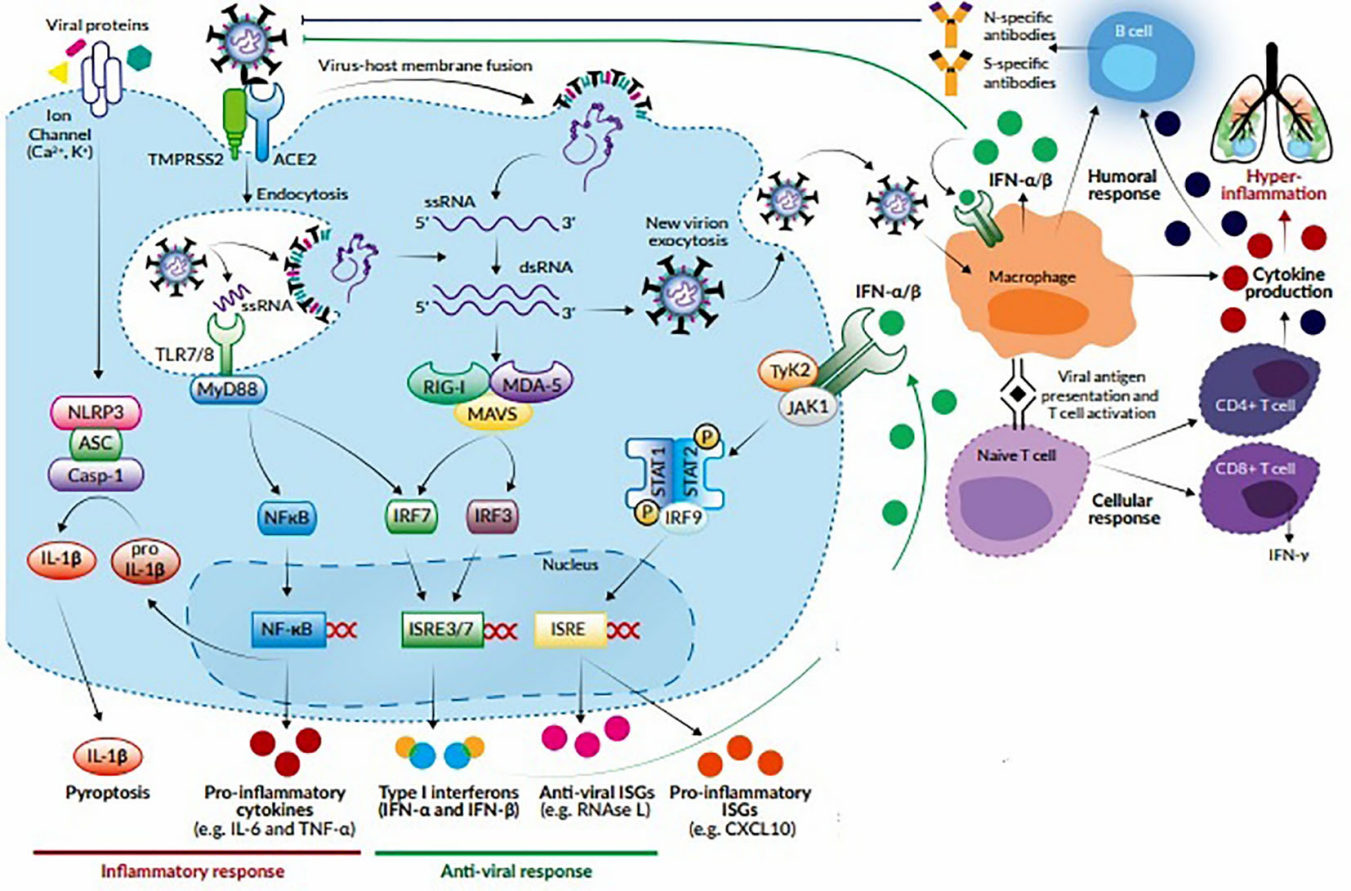
Host response to SARS-CoV-2. The virus attaches to ACE2 receptors and enters the target cell by membrane fusion. Upon entry, the virus is recognized by innate immune receptors TLR7/8, cytosolic RNA sensors RIG-I/MDA-5, and the inflammasome sensor NLR family pyrin domain-containing-3 (NLRP3). This leads to the activation of NF-кB and IRF3/7 and the subsequent production of pro-inflammatory cytokines (e.g., IL-1β, IL-6, and TNF-α) and type I IFNs, respectively. Cytokines released by infected cells modulate the adaptive immune response by causing recruitment and activation of macrophages, B cells, and T cells which facilitate elimination of the virus. However, an unbalanced immune response can cause massive release of pro-inflammatory cytokines, leading to a cytokine storm which is responsible for the severe clinical manifestations of COVID-19.

The entry of virus into the host cells triggers stimulation of innate immune response *via* antigen presenting cells (APCs) like dendritic cells and macrophages, which represent the first line of defence against viruses. APCs have pattern recognition receptors (PRRs), such as, Toll-like receptors (TLRs), NOD-like receptors (NLRs), RIG-I-like receptors (RLRs), and melanoma differentiation-associated protein 5 (MDA5) present at various locations like plasma membrane, endosomal membrane, lysosomes, and cytosol (Li et al., 2020). They recognize various structural components of the virus, such as, nucleic acids, carbohydrate moieties, glycoproteins, lipoproteins, and dsRNA and induce a signaling cascade to produce the immune system effectors. The APCs present the viral antigenic peptides to the CD8+ T cells in association with major histocompatibility complex (MHC) class I. The CD8+ T cells get activated, undergo clonal expansion and develop into virus-specific effector and memory T cells. With their perforin and granzymes, CD8+ T cells lyse the virus-infected cells and induce apoptosis. In addition, there occurs an upregulation of natural killer (NK) cell activation and production of pro-inflammatory cytokines *via* the nuclear factor kappa B (NF-κB) and interferon regulatory factor 3 (IRF3) signaling pathways. This leads to further recruitment of neutrophils and monocytes to the site of infection and activation of several other pro-inflammatory cytokines (Astuti, 2020; Li et al., 2020). During an infection, activation and priming of innate and adaptive immune responses result in pathogen clearance and recovery. However, SARS-CoV-2 causes suppression of host’s innate immune response by inhibiting certain signaling pathways and thus, evades detection by the immune system, leading to a more severe disease and fatal outcomes (Felsenstein et al., 2020). It has been postulated that SARS-CoV-2, like SARS-CoV, alters the ubiquitination and degradation of RNA sensors (RIG-I and MDA5) and inhibits the activation of mitochondrial antiviral-signaling protein (MAVS), thereby preventing the activation and nuclear translocation of IRF3 in response to activated RNA sensors. Moreover, SARS-CoV2 inhibits tumor necrosis factor (TNF) receptor–associated factors (TRAF) 3 and 6, which are crucial for the induction of IRF-3/7 in response to TLR3/7 and NFκB signaling pathways. It also inhibits the phosphorylation of Janus kinase/signal transducers and activators of transcription (JAK/STAT) transcription factor and blocks type I/III interferon (IFN) signaling pathways (Kindler et al., 2016). These mechanisms allow the virus to replicate evading the innate antiviral responses and induce the production of cytokines required for recruitment of adaptive immune cells. The transition between innate and adaptive immune responses is critical for the clinical course of COVID-19. This phase determines whether the immune regulatory events will culminate in protective immunity or an exacerbated immune response. The protective immunity is T cell mediated, with CD8+ T cells eliminating the virus-infected cells and CD4+ T cells helping the B cells to produce neutralizing antibodies and orchestrating the response of other immune cells. The T cells account for 80% of the infiltrating cells in SARS-CoV-2 infection. However, a dysregulated T cell response can result in immunopathology leading to exaggerated cytokine release and a cytokine storm (Cao, 2020; Tay et al., 2020). This condition is characterized by increased secretion of pro-inflammatory cytokines, such as interleukin (IL)-1β, IL-2, IL-6, IL-7, IL-8, IL-9, IL-10, and IL-17; granulocyte-macrophage colony stimulating factor (GM-CSF); TNF-α, IFN-γ and IFN-γ inducible protein 10 (IP10); monocyte chemoattractant protein 1 (MCP-1); macrophage inflammatory protein- 1 alpha and -1 beta (MIP-1α and -1β); chemokines like CC chemokine ligand 2 (CCL2), CCL3, and CCL5; and C-X-C motif chemokine ligand 8 (CXCL8), CXCL9, and CXCL10. The cytokine storm induces a hyperinflammatory state causing acute lung injury and various complications like ARDS, respiratory failure, shock, disseminated intravascular coagulation, multiorgan failure and death (Qin et al., 2020; Xu Z. et al., 2020). This complex cascade of inflammatory response triggers platelet activation, endothelial dysfunction, and vascular stasis. Recent studies suggest that COVID-19 induces a hypercoagulable state that may predispose to venous and arterial thromboembolic events and worsened outcomes (Abou-Ismail et al., 2020). The humoral immune response is critical for virus clearance and preventing reinfection. SARS-CoV-2 elicits a robust B cell response, as evidenced by detection of virus-specific neutralizing antibodies in most cases following infection. Seroconversion occurs between 7 and 14 days after symptom-onset, and antibody titers persist in the weeks following virus clearance (Vabret et al., 2020). The RBD of S protein is highly immunogenic, and antibodies against this domain can block virus interaction with the host ACE2 receptors and thus, prevent virus entry (Ju et al., 2020). The subepithelial dendritic cells and macrophages recognize the viral proteins and present them to CD4+ T cells in association with MHC class II, which induces differentiation of these T cells into Th1, Th17, and memory T follicular helper (T_FH_) subsets. The T_FH_ cells induce the conversion of B cells to plasma cells and promote the production of virus-specific IgM, IgA, and IgG antibodies (Cao, 2020). Like other viral infections, the initial antibody response in COVID-19 is predominantly IgM, which is transient and short-lived and soon gets replaced by IgG antibodies. The latter has a longer half-life and lower molecular weight, which enable it to confer long-term protection and effective tissue penetration. However, different patterns of IgM and IgG seroconversion have been observed, such as synchronous seroconversion, IgM seroconversion preceding that of IgG, and IgM seroconversion later than that of IgG. Secretory IgA plays a crucial role in mucosal immunity by neutralizing the virus and preventing its attachment to the mucosal epithelium (Long et al., 2020). The proposed host immune response to SARS-CoV-2 has been shown in **Figure 1**.

## Potential Therapeutic Candidates: Novel Virus and Novel Targets

**Figure 2:**
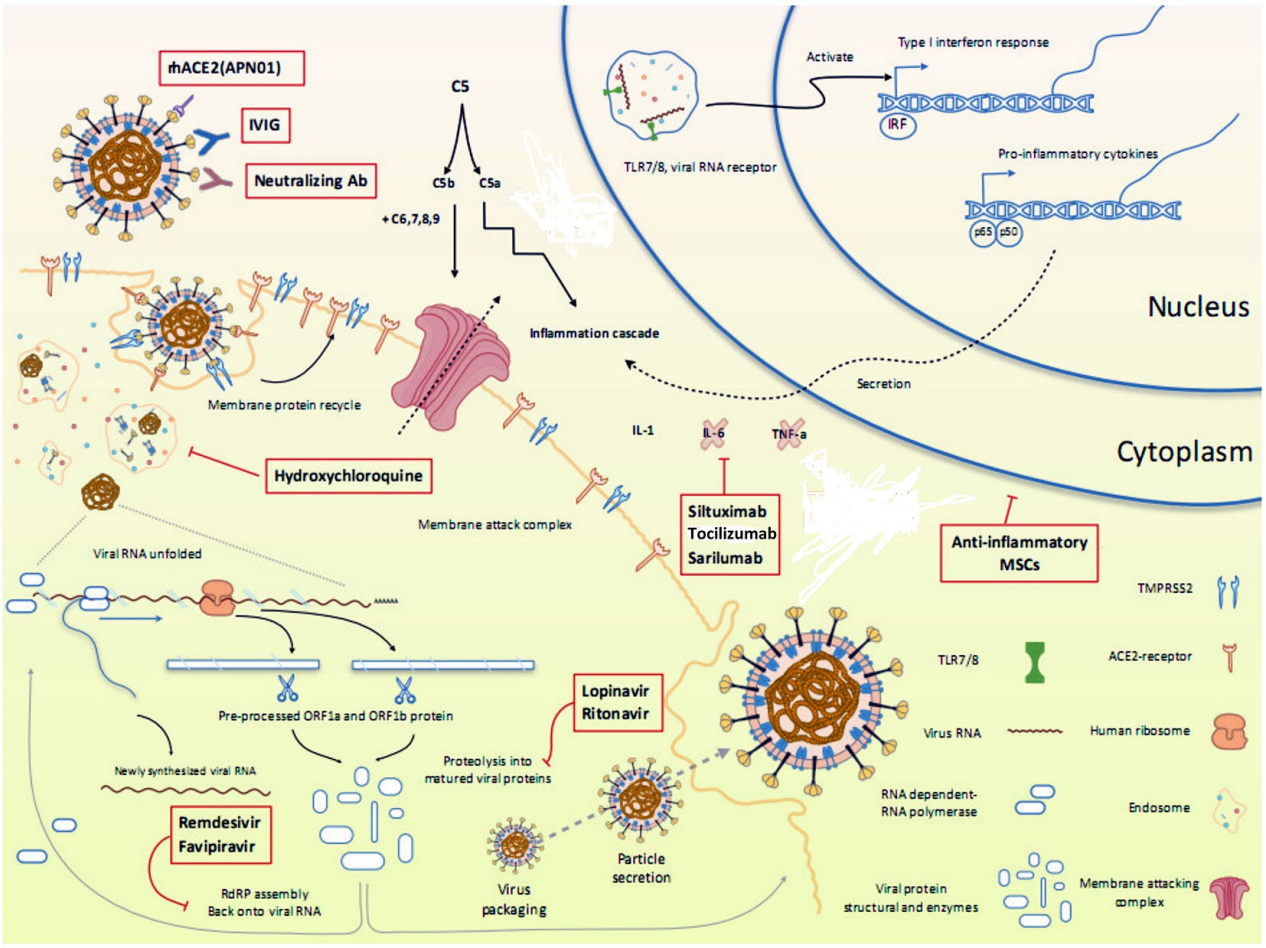
Overview of various repurposed and investigational drugs undergoing clinical trials against COVID-19 in the context of viral life cycle and host immune response. Reproduced from Tu et al. (2020).

Currently, there are no clinically proven antiviral drugs or biologics for the treatment of COVID-19 patients. A protocol issued by National Health Commission of the People’s Republic of China states that optimized symptomatic management, together with respiratory support should be the mainstay of treatment (Guo et al., 2020). Most existing data on antiviral therapy for COVID-19 are derived from related coronaviruses, such as, SARS-CoV (2003), and MERS-CoV (2012) and non-coronaviruses such as Ebola virus. How well these data can be extrapolated to SARS-CoV-2 remains unclear. Moreover, a lack of pharmacokinetic/pharmacodynamic or clinical data comparing achievable exposures with treatment outcomes further questions the clinical relevance of *in vitro* activity of antiviral drugs, which may vary widely and therefore, should be compared cautiously. Since the onset of this pandemic, several studies emphasizing the therapeutic benefits of a wide range of antiviral drugs and biologics have been published in medical literature. However, a thorough analysis of these drugs is warranted to ascertain whether the existing evidence supports the currently proposed management strategies. An overview of various repurposed and investigational drugs undergoing clinical trials against COVID-19 has been depicted in **Figure 2** (Tu et al., 2020). There are more than 300 ongoing clinical trials evaluating the safety and efficacy of these drugs. The major proposed therapeutic candidates that seem promising for the treatment of COVID-19 are summarized in **Table 1**.

**Table 1:** Summary of potential therapeutic candidates for COVID-19.

Drug class	Drug name	Previous targets/viruses	Mechanism of action	Trial identifier	Country (completion date)	Phase
Antivirals	Remdesivir	HCV, Marburg, Ebola viruses	Adenosine analog, incorporates into the nascent viral RNA chain, causing delayed chain termination during RNA replication	NCT04292730 NCT04292899 NCT04280705 2020-000936-23 NCT04257656 NCT04252664	USA (June 2020) USA (June 2020) USA (May 21, 2020) France (not available) China (Apr. 10, 2020) China (Apr. 27, 2020)	III III III III III III
	Lopinavir/ritonavir	HIV-1	3C-like protease inhibitor	NCT04330690 NCT04328012 NCT04315948 CTRI/2020/04/024773	Canada (May 18, 2022) USA (Apr.1, 2021) France (Mar. 2023) India (Apr. 2021)	II II III III
	Umifenovir	Influenza A, HCV,HBV, Ebola virus, poliovirus	S protein/ACE2, membrane fusion inhibitor	NCT04260594 NCT04255017 NCT04252885 NCT04286503	China (Dec. 30, 2020) China (July 1, 2020) China (May 31, 2020) China (Feb. 28, 2021)	IV IV IV IV
	Danoprevir/ritonavir	HCV 1−6	Protease inhibitor	NCT04291729 NCT04303299 NCT04304053	China (Mar. 19, 2020) Thailand (Mar. 30, 2021) Spain (June 15, 2020)	IV III III
	Favipiravir	Influenza A, arenaviruses, bunyaviruses, filoviruses	RNA dependent RNA polymerase inhibitor	NCT04336904 CTRI/2020/05/025114	Italy (July 2020) India (Aug. 2020)	III III
	Interferon	HCV, HBV, HIV (in combination with HAART	Inhibit viral replication through secretion of cytokines. IFNβ is a more potent inhibitor of coronaviruses than IFNα	NCT04276688 NCT04315948 NCT04343768 NCT04293887	Hong Kong (Mar. 31, 2020) France (March 2023) Iran (Apr. 27, 2020) China (June 30, 2020)	II III II I
Antiparasitic drugs	Nitazoxanide	*B. hominis*, *C. parvum*, *G. lamblia*, *H. nana*, *E. histolytica*, *A. lumbricoides*, HBV, HCV, rotavirus, norovirus	Interferes with host-regulated pathways involved in viral replication	NCT04341493	Mexico (Dec.30, 2020)	IV
	Ivermectin	*S. scabiei, O. volvulus, S. stercoralis, Loa loa, W. bancrofti, T. trichura*, Zika, dengue, yellow fever, West Nile, Hendra, Newcastle, VEE, chikungunya, HIV-1, BK polyoma, pseudorabies	Inhibits IMPα/β1-mediated nuclear import of viral proteins	NCT04373824 NCT04392713	India (July 25, 2020) Pakistan (July 2020)	NA NA
Immunomodulators and biologics	Hydroxychloroquine and azithromycin (HCQ/AZ)	HCQ: Uncomplicated *P. falciparum, P. vivax, P ovale, P. malariae*, Q fever, *T. whipplei* AZ: Bacterial infections, Zika, Ebola	Increases endosomal pH, prevents viral fusion, reduces release of pro-inflammatory cytokines, inhibits terminal glycosylation of ACE2, and prevents viral entry.	NCT04345692 NCT04332991 NCT04329832 NCT04340544 NCT04329611 NCT04336332 NCT04408456 CTRI/2020/04/024904	USA (Dec. 31, 2021) USA (July 2021) USA (Dec. 31, 2021) Germany (Sept. 22, 2021) USA (Aug. 31, 2020) USA (Apr. 30, 2021) India (June 30, 2020) India (May 2021)	III III II III III II III III
	Tocilizumab	RA, JIA, GCA, CRS	mAb against IL-6 receptor- prevents cytokine storm	NCT04320615 NCT04317092 NCT04332913	France (Sept.30, 2020) Italy (Dec.19, 2022) Italy (Mar.31, 2021)	III II −
	Sarilumab	RA (DMARD)	mAb against IL-6 receptor- inhibits IL-6-mediated signaling	NCT04327388 NCT04315298	Italy, France, Spain, Canada (Aug. 2020) Italy, France, Spain, Canada (Aug. 31, 2020)	III II/III
	Bevacizumab	Malignancies (colon, breast, lung, kidney, brain, ovary), diabetic retinopathy, ARMD	mAb against VEGF- decreases vascular permeability	NCT04275414	China (May 2020)	II/III
	Meplazumab	Chloroquine resistant *P. falciparum*	mAb against CD147- inhibits viral replication and CyPA-induced T cell chemotaxis	NCT04275245	China (Dec.31, 2020)	I/II
	Itolizumab	Chronic plaque psoriasis	Anti-CD6 IgG1 mAb- suppresses pro-inflammatory cytokines and cytokine storm	CTRI/2020/05/024959	India (July 7, 2020)	II
	Anakinra	RA (DMARD), NOMID, MAS, HLH	IL-1 receptor antagonist	NCT04330638 NCT04324021	Belgium (Dec. 2020) Sweden (Sept. 2020)	III II/III
Cellular therapies	Mesenchymal stem cells	Autoimmune diseases (SLE, Chron’s disease, GVHD), CRS	Immunomodulatory activity- prevents cytokine storm. Antimicrobial activity due to production of AMPs like cathelicidin, defensin, and lipocalin, hepcidin.	NCT04288102 NCT04315987	China (July 10, 2020) Brazil (Aug. 2020)	II II
	NK cells	Multiple myeloma, AML, GBM	Cytokine secretion, perforin-granzyme, and death receptor medicated cytolysis	NCT04280224 NCT04324996	China (Dec. 30, 2020) China (Sept. 30, 2020)	I I/II
Immunotherapy	Convalescent plasma	Ebola, MERS-CoV, H1N1, H5N1, measles, Junin, VZV, CMV, parvovirus B19	Viral inactivation through neutralizing antibodies	NCT04332835 NCT04425915	Colombia (Dec. 31, 2020) India (May 30, 2021)	II/III III
Adsorbent therapy	CytoSorb therapy	Septic shock, SIRS, RAM, liver failure	Adsorbs circulating cytokines prevents cytokine storm	NCT04324528	Germany (Nov.26, 2020)	−

HCV, hepatitis C virus; HBV, hepatitis B virus; HIV, human immunodeficiency virus; HAART, highly active antiretroviral therapy; VEE, Venezuelan equine encephalitis, RA, rheumatoid arthritis; JIA, juvenile idiopathic arthritis; GCA, giant cell arteritis; CRS, cytokine release syndrome; DMARD, disease modifying antirheumatic drugs; ARMD, age-related macular degeneration; NOMID, neonatal onset multisystem inflammatory disease; MAS, macrophage activation syndrome; HLH, hemophagocytic lymphohistiocytosis; SLE, systemic lupus erythematosus; GVHD, graft versus host disease; AML, acute myeloblastic leukemia; GBM, glioblastoma multiforme; VZV, Varicella Zoster virus; CMV, cytomegalovirus; SIRS, systemic inflammatory response syndrome; RAM, rhabdomyolysis associated myoglobinemia; ACE2, angiotensin converting enzyme 2; IMP, importin; IFN, interferon; TLR, Toll-like receptor; IL, interleukin; VEGF, vascular endothelial growth factor.

### Antivirals

#### Remdesivir

Remdesivir (Veklury; Gilead Sciences, Inc.) is an analog of adenosine triphosphate, which incorporates into the nascent viral RNA chains and results in delayed chain termination during replication of viral RNA. It has broad-spectrum antiviral activity against several RNA viruses including Ebola, Marburg, MERS-CoV, SARS-CoV, respiratory syncytial virus (RSV), Nipah virus, and Hendra virus and has demonstrated prophylactic and therapeutic efficacy against coronaviruses (Gordon et al., 2020). Use of remdesivir in SARS-CoV–infected mice resulted in reduced viral loads and improved disease outcomes. Recently, the drug has been shown to possess *in vitro* activity against SARS-CoV-2. Remdesivir seems to possess a favorable safety profile, as evidenced in 500 participants, including healthy volunteers and patients who received remdesivir for Ebola virus disease (Mulangu et al., 2019). Its prophylactic and therapeutic efficacy was demonstrated in a rhesus macaque model of MERS-CoV infection, in which prophylactic administration of remdesivir 24 hours prior to MERS-CoV inoculation completely prevented clinical disease, inhibited viral replication, and prevented the development of pulmonary lesions. Therapeutic administration of the drug 12 hours post-inoculation reduced the severity of clinical symptoms, attenuated viral replication, and decreased the pulmonary lesions (de Wit et al., 2020). Gilead sciences, in a recent case series, considered compassionate-use of remdesivir in 53 COVID-19 patients with severe disease and reported that 68% of the cases showed clinical improvement after a median follow-up of 18 days, with mortality of 13% and a favorable safety profile (Grein et al., 2020). The findings were, however, not compared with a control group that received only standard care. At present, there are six ongoing clinical trials evaluating the safety and efficacy of remdesivir in adult patients diagnosed with COVID-19 (moderate/severe disease): two initiated by Gilead Sciences, one by National Institute of Allergy and Infectious Diseases (NIAID), one by INSERM (France), and two by China-Japan Friendship Hospital. All these clinical trials are currently in phase III. Formal recommendations regarding the use of remdesivir can be made once these trials come up with some conclusive evidence.

#### Lopinavir/Ritonavir

Lopinavir/ritonavir (LPV/r; Kaletra) is a combination of protease inhibitors used for the treatment of HIV infection. Ritonavir is also a potent inhibitor of cytochrome P450, a class of enzymes responsible for metabolism of lopinavir, and the co-administration augments the plasma levels of lopinavir, improving its antiviral activity (Molla et al., 2002). LPV/r has demonstrated in-vitro antiviral activity against SARS-CoV and MERS-CoV. Since this combination was not specifically formulated for treatment of coronavirus infections, this alone may not demonstrate a significant advantage over placebo in reducing viral load (Yao et al., 2020). A clinical trial involving 199 patients with laboratory-confirmed SARS-CoV-2 infection reported that LPV/r combination did not offer any clinical benefit over the standard management (Cao et al., 2020). There are several ongoing clinical trials comparing the efficacy of LPV/r alone and in combination with other drugs like umifenovir, carrimycin, danoprevir/ritonavir, interferon, xiyanping, and traditional Chinese medicines. LPV/r in combination with IFN-β1b reduced MERS-CoV viral load and improved lung pathology in a marmoset model (Yao et al., 2020). However, Sheahan et al. (2020) reported that combining LPV/r with IFN-β did not significantly augment the antiviral activity of the latter against MERS-CoV. In an open label clinical trial involving hospitalized SARS patients, LPV/r in combination with ribavirin was found to decrease the mortality rate and requirement of ventilator support compared to the control group (median, 6 days versus 11 days; 95% CI, −9–0) (Cao et al., 2020). Thus, considering the therapeutic benefits in the treatment of SARS and MERS, the safety and efficacy of LPV/r based combination regimen in the treatment of COVID-19 needs to be evaluated.

#### Umifenovir

Umifenovir (Arbidol, Pharmstandard Ltd.) is a fusion inhibitor that interacts with viral hemagglutinin and prevents the fusion of viral envelope with host cell membrane. The drug is currently licensed for use only in Russia and China for the treatment and prophylaxis of influenza and other respiratory viral infections. Umifenovir has a broad-spectrum antiviral activity due to its dual action as direct-acting antiviral and host-targeting agent. It has been found to be active against several enveloped and non-enveloped RNA and DNA viruses, including Chikungunya virus, Zika virus, foot-and-mouth disease virus, Lassa virus, Ebola virus, HSV, HBV, HCV, chikungunya virus, reovirus, Hantaan virus, and coxsackie virus B5 (Blaising et al., 2014; Kadam and Wilson, 2017). It also inhibits clathrin-mediated exocytosis and intracellular trafficking by interacting with the cell membrane (Blaising et al., 2013). Considering its unique mechanism of action, umifenovir alone and in combination with antiretroviral drugs is currently being investigated for treatment and prophylaxis of COVID-19. However, a retrospective study by Lian et al., involving 81 COVID-19 patients showed that umifenovir did not shorten the SARS-CoV-2 negativity time or improve the prognosis in non-ICU patients compared to the supportive treatment (Lian et al., 2020). There are currently four ongoing clinical trials of umifenovir for COVID-19 treatment: one in comparison with the basic treatment^6^, and the other three comparing the effects in combination with oseltamivir^7^, lopinavir/ritonavir^8^, and carrimycin.^9^

#### Favipiravir

Favipiravir (Avigan, Toyama Chemical Co. Ltd.), a modified pyrazine analog, is a potent inhibitor of viral RNA dependent RNA polymerase (RdRp) approved in Japan since 2014, for the treatment of oseltamivir-resistant cases of influenza (Furuta et al., 2017). Besides influenza A and B, it has been found to be effective against avian influenza. It has also been investigated for the treatment of infections caused by Ebola virus, Lassa virus, and now, SARS-CoV-2 (Du and Chen, 2020). Favipiravir is a prodrug that gets metabolized to an active form favipiravir-ribofuranosyl-5′-triphosphate (favipiravir-RTP), which selectively binds to RdRp and inhibits viral replication. In contrast to the existing antivirals against influenza that primarily block the entry and exit of the virus from cells, favipiravir’s novel mechanism of action allows its active form to get incorporated into the nascent RNA strand, thus preventing strand elongation and viral proliferation. The drug has an oral bioavailability of 97.6 and is 54% plasma protein-bound with an elimination half-life of 2–5 hours (Du and Chen, 2020). The RdRp gene of SARS-CoV-2 is structurally similar to that of SARS-CoV and MERS-CoV, as revealed by genome sequencing (Cascella et al., 2020). A clinical trial (ChiCTR2000029600) conducted in Shenzhen, China reported that COVID-19 patients who received favipiravir demonstrated significantly shorter viral clearance time and higher improvement in chest imaging, compared to the control group (4 days, 91.4% versus 11 days, 62%) (Cai et al., 2020). In another multi-centre randomized trial (ChiCTR2000030254), treatment with favipiravir was found to be beneficial for COVID-19 patients with diabetes and/or hypertension as evidenced by decreased time-to-relief for fever and cough. Also, seven days clinical recovery rate increased from 55.9 to 71.4% (Chen C. et al., 2020). These studies indicate that favipiravir can be a safe and effective treatment option for COVID-19. The drug is currently undergoing phase III clinical trial, which is expected to be completed by July 2020.

### Interferon

IFNs are a family of inducible cytokines produced by various cell types in response to viral infections. IFNs exert their actions through pattern recognition receptors (PRRs), which are largely species specific. Of particular interest are the type 1 IFNs (viral IFNs), which are secreted by the plasmacytoid dendritic cells and are among the first cytokines produced during a viral infection. IFN-I comprises of several subtypes (α, β, ϵ, ω, and κ) (Samuel, 2001), which exert their actions after binding with interferon-α/β receptor (IFNAR). Ligand binding induces phosphorylation of the receptor and activation of signal transducers and several transcriptional factors such as STAT1 and STAT2. These form complexes that are translocated to the nucleus, where they activate interferon-stimulated genes (ISG). ISGs include PRRs, IRFs, and members of the JAK-STAT signaling pathway, which sensitize the cell to pathogens, and play a prominent role in inflammation, antiviral innate signaling, immunomodulation, and interfere with several steps of viral replication (Schneider et al., 2014). Thus, IFN-I plays a vital role in antiviral immunity. Because of their immunomodulatory and antiviral properties, they are often evaluated for the treatment of several emerging viral infections. SARS-CoV-2 bears a close resemblance with other members of the *Coronaviridae* family such as MERS-CoV and SARS-CoV and exhibits similar properties, despite differences in their epidemiology, pathology, and several of their structural proteins. Numerous *in vivo* and *in vitro* studies have evaluated the role of IFN-I in the treatment of MERS-CoV and SARS-CoV, either alone or in combination with lopinavir/ritonavir (Chan et al., 2015), ribavirin (Omrani et al., 2014), remdesivir, corticosteroids, and IFN-γ (Sainz et al., 2004). Though both IFN-α and-β have demonstrated efficacy *in vitro* and succeeded in certain animal models, they failed to improve the disease in humans. Such difference in therapeutic responses could be attributed to IFN signaling pathway used by the viruses, limited number of study subjects, varied experimental settings or clinical conditions, and IFN-subtype diversity. Studies have shown that IFNβ, particularly the β1 subtype (IFNβ1b or IFNβ1a), is a more potent inhibitor of coronaviruses than IFNα and thus appears to be more relevant in the treatment coronavirus infections (Stockman et al., 2006). In the lungs, IFNβ1 stimulates the secretion of anti-inflammatory adenosine and promotes maintenance of endothelial barrier function by up-regulating CD73 in pulmonary endothelial cells. This can be a possible explanation to the reduction of vascular leakage in ARDS with IFNβ1a treatment (Bellingan et al., 2014). The timing of IFN-I administration plays a critical role, with positive effects being observed early in the course of infection while delayed administration failed to inhibit viral replication (Channappanavar et al., 2019). Based on previous knowledge, it has been hypothesized that SARS-CoV and MERS-CoV are able to disrupt the interferon signaling pathway probably through involvement of ORF6 and ORF3b (Kopecky-Bromberg et al., 2007). However, due to the truncated nature of ORF6 and ORF3b proteins in SARS-CoV-2, they may have lost their anti-interferon activities. This could be a possible explanation for SARS-CoV-2 displaying substantial *in vitro* sensitivity to IFN-I. Thus, IFN-I is expected to be more promising for the treatment of COVID-19 than for SARS (Lokugamage et al., 2020). The assumption is further supported by the fact that IFNα2b sprays minimise the infection rate of SARS-CoV-2 and can be used prophylactically against the virus (Shen and Yang, 2020). All these facts support that IFN-I might be a safe and effective treatment against SARS-CoV-2. The knowledge acquired from studies on MERS-CoV or SARS-CoV indicates that for optimum effects and better safety profile, IFN-I should be administered early in the course of infection. In the later phases, the overwhelming inflammatory response caused by massive release of cytokines might call for anti-interferon drugs to mitigate the pathology. In China, the guidelines for the treatment of COVID-19 recommend administration of 5 million units of IFNα by vapor inhalation twice a day, in combination with ribavirin (Dong et al., 2020). Vapor inhalation offers the advantage of specifically targeting the respiratory tract. The efficacy of IFN-I can be further improved if given in combination with lopinavir/ritonavir, ribavirin, or remdesivir because of the efficacy of such combinations observed *in vitro* against other coronaviruses (Sheahan et al., 2020). Further research on IFN-based treatment is expected in near future, which should give more accurate information on the efficacy of this therapy and possible outcomes.

### Ivermectin

Ivermectin (Stromectol; Merck & Co., Inc.) is a broad spectrum anthelmintic agent belonging to class of avermectins and is derived from the soil bacterium *Streptomyces avermitilis*. It’s selective and high affinity binding with glutamate-gated chloride channels in nerve and muscle cells of nematode, increases the permeability of the cell membrane to chloride ions, resulting in hyperpolarization of cells and paralysis and death of the parasite. It is 93.2% plasma protein-bound and has a half-life of 18 hours following oral administration. The drug was originally launched by Merck Laboratories in 1987 for use against onchocerciasis (river blindness) as a part of the Onchocerciasis Control Programme in West Africa. Subsequently, the drug was approved for the treatment of a number of human parasitic infections including strongyloidiasis, ascariasis, trichuriasis, enterobiasis, lymphatic filariasis, and scabies in several countries (Australia, France, Japan, the Netherlands, USA, etc) (Ikeda, 2003). Besides its anti-parasitic action, several studies have demonstrated the potent antiviral activity of ivermectin against a broad range of viruses *in vitro* (Caly et al., 2020). It has been shown to inhibit the interaction between the HIV-1 integrase protein (IN) and the importin (IMP) α/β1 heterodimer, causing inhibition of HIV-1 replication (Wagstaff, 2012). Ivermectin has also been reported to limit infections caused by several RNA viruses (dengue viruses 1-4, West Nile Virus, Venezuelan equine encephalitis virus, and influenza virus) and DNA virus (pseudorabies virus) (Wagstaff, 2012; Caly et al., 2020). Studies have found that host cell division might be affected during SARS-CoV infection, due to a signal-dependent nucleocytoplasmic shutting of the viral nucleocapsid protein involving IMPα/β1 (Timani, 2005; Wulan, 2015). The antiviral activity of the STAT1 transcription factor is blocked by SARS-CoV accessory protein ORF6, which causes sequestration of IMPα/β1 on the rough endoplasmic reticulum/Golgi membrane (Frieman, 2007). Considering ivermectin’s inhibitory action on IMPα/β1-mediated nuclear import, it is presumed to be effective against SARS-CoV-2. Caly et al. (2020) studied the antiviral activity of ivermectin against SARS-CoV-2 and observed that a single treatment with ivermectin was able to cause ∼5000-fold reduction of virus titre at 48 h in Vero/hSLAM cell culture. Ivermectin has a favorable safety profile in humans with high dose therapy considered as safe as the standard low-dose regimen. However, the therapeutic benefits from multiple drug dosing need to be evaluated in COVID-19 patients. An effective antiviral drug given early in the course of infection can help reduce the viral load and prevent disease progression while limiting person-person transmission. Ivermectin’s unique antiviral action combined with a favorable safety profile allows it for further consideration as a possible treatment option in COVID-19.

### Immunomodulators and Biologics

#### Hydoroxycholoquine and Azithromycin

Hydroxychloroquine (HCQ) (Plaquenil; Sanofi‐Synthelabo Inc.) is an aminoquinoline like chloroquine and is indicated for the treatment of uncomplicated malaria, prophylaxis of malaria in places without chloroquine resistance, chronic discoid lupus erythematosus, systemic lupus erythematosus, and rheumatoid arthritis. In addition, HCQ has been found to be effective against intracellular bacteria such as *Coxiella burnetii* (Raoult et al., 1990) and *Tropheryma whipplei* (Boulos et al., 2004). HCQ has also been shown to possess antiviral properties and is already being used in clinical trials for the treatment of HIV infection. It increases endosomal pH which prevents viral fusion and entry into the host cells, inhibits antigen processing and presentation, blocks dimerization of major histocompatibility complex (MHC) class II, and reduces host inflammatory response by decreasing the release of cytokines like IL-1 and TNF-α. HCQ inhibits terminal glycosylation of ACE2 receptor, the main portal of entry for SARS-CoV and SARS-CoV-2. Non-glycosylated ACE2 interacts less efficiently with the viral spike protein, thus preventing viral entry (Colson et al., 2020). Several studies have proposed that repurposing of approved drugs such as chloroquine, HCQ, azithromycin, metformin, losartan, and simvastatin could be useful in the treatment of COVID-19. Clinical trials from China have shown the efficacy of chloroquine in the treatment of COVID-19 patients, as evidenced by subsidence of fever, improvement of radiological findings, and delay in disease progression. Azithromycin (AZ) is a macrolide antibiotic that has demonstrated *in vitro* activity against Zika and Ebola viruses (Bosseboeuf et al., 2018). Several authors have mentioned a synergistic effect of HCQ/AZ combination in the treatment of COVID-19. An open label non-randomized clinical trial from France showed that COVID-19 patients treated with 600 mg of HCQ daily had a significant reduction in viral carriage at day 6 post-inclusion, with 70% of the patients having a negative PCR test result compared to only 12.5% in the untreated control group. Moreover, patients who were treated with a combination of HCQ and AZ (500 mg on day 1, followed by 250 mg daily for the next four days) showed complete virological cure at day 6 post-inclusion compared to 57.1% in the group that received HCQ alone (Gautret et al., 2020a). Another study from France claimed that patients who received a combination of HCQ and AZ had a significant clinical improvement as evidenced by a rapid fall in viral load, with 83% tested negative by quantitative PCR on day 7 and 93% on day 8. Virus cultures of respiratory samples were negative in 97.5% patients on day 5 (Gautret et al., 2020b). However, the apparent beneficial effects of HCQ in the treatment of COVID-19 have been completely negated by a pilot study from China, where no significant differences in outcomes were observed between HCQ-treated group and the control group (Chen J. et al., 2020). A large observational study in hospitalized COVID-19 patients in the US also showed that treatment with HCQ was not associated with significant clinical benefits and has no influence on intubation or death (Geleris et al., 2020). Furthermore, the use of HCQ alone or in combination with AZ is not free from hazards. Both these drugs are associated with an increased risk of QT_c_ prolongation, torsades de pointes, ventricular tachycardias, and gastrointestinal side effects. It has been observed that patients receiving a five-day course of AZ had an increased risk of sudden cardiac death with a hazard ratio of 2.71 (Ray et al., 2012). Considering the cumulative adverse effects of HCQ and AZ on cardiac conduction, it is advised to have baseline and follow-up ECG monitoring, along with careful consideration for other concomitant medications known to prolong the QT_c_ interval, if this combination has to be used. Guidelines published by the Infectious Disease Society of America mentioned that despite a higher proportion of clinical improvement in the HCQ group, the beneficial effect of HCQ on viral clearance or disease progression cannot be judged by the currently available evidence due to certain drawbacks such as small sample sizes, ill-defined patient selection criteria, co-interventions, and methodological limitations (Bhimraj et al., 2020). Moreover, none of the studies have addressed patient-relevant outcomes like mortality, rate of disease progression to ARDS, and need for mechanical ventilation. Also, the mortality rate among patients receiving HCQ/AZ combination was not compared with an untreated cohort. Though studies have claimed that patients receiving HCQ and AZ experienced less virologic failure (43% pooled virologic failure) as compared to historical controls (100% virologic failure) (Gautret et al., 2020b; Molina et al., 2020), such comparison lacks certainty because of unmeasured confounding and selection bias. Furthermore, these studies have relied mainly on intermediary outcomes such as reduction in development of pneumonia, and less hospital or ICU admission to ascertain therapeutic benefits, which raise question on their precision and feasibility. Therefore, a RCT should be the ideal approach for determining the therapeutic effects of HCQ in COVID-19 patients.

#### Monoclonal Antibodies

##### Tocilizumab

The leading cause of mortality in COVID-19 is respiratory failure from ARDS. A cytokine profile resembling secondary hemophagocytic lymphohistiocytosis (HLH), characterized by a fulminant and fatal hypercytokinemia with multiorgan failure is associated with COVID-19. There is a massive and uncontrolled release of pro-inflammatory cytokines like IL-2, IL-6, G-CSF, IP10, MCP-1, MIP-1-α and TNF-α (Mehta et al., 2020; Xu Z. et al., 2020). A recent retrospective study involving 150 confirmed COVID-19 cases from Wuhan, China, revealed that elevated levels of serum ferritin and IL-6 were independent predictors of fatality, probably due to virally driven hyperinflammation (Ruan et al., 2020). Tocilizumab (Actemra, Roche) is a humanized monoclonal antibody against the interleukin-6 receptor (IL-6R) approved for the treatment of seriously ill COVID-19 patients with elevated IL-6 by the National Health Commission of China. Xu X. L. et al. (2020) observed the effects of tocilizumab in 21 COVID-19 patients with severe disease, in addition to routine therapy, and reported significant therapeutic benefits as evidenced by subsidence of fever and other symptoms within a few days and improvement of oxygen saturation in 75% of patients. There were no obvious treatment-related adverse reactions. In another report from China, a case of COVID-19 with pre-existing multiple myeloma was successfully treated with tocilizumab, highlighting its potential therapeutic benefits in the treatment of COVID-19 patients (Zhang et al., 2020). On March 26, 2020, the drug entered phase III clinical trial for the treatment of COVID-19 pneumonia.

##### Bevacizumab

The main contributory factors for increased mortality in COVID-19 patients are acute lung injury (ALI) and ARDS, brought about by a cytokine-mediated hyperinflammatory response. Pulmonary edema is the key detrimental feature of ALI/ARDS. COVID-19 is associated with more exaggerated pulmonary mucus exudation than SARS as revealed by autopsy (Xu et al., 2020). Pulmonary imaging and histopathological examination also support similar findings. However, specific pharmacotherapy to combat this pathology is lacking. Vascular endothelial growth factor (VEGF) is one of the most potent inducers of increased vascular permeability in COVID-19-affected lungs, causing fluid extravasation and pulmonary edema. Expression of VEGF is induced by hypoxia through activation of Prolyl hydrolases (PHD)-hypoxia inducible factor (HIF)-1 pathway, which upregulates transcription of VEGF. Therefore, blockade of VEGF signaling pathway might help in reducing inflammation and improving tissue perfusion in patients with severe COVID-19. Bevacizumab (Avastin; Genentech Ltd.) is a recombinant humanized monoclonal antibody targeted against VEGF and is currently recommended for the treatment of malignancies (colorectal, lung, breast, renal, brain, and ovarian), age-related macular degeneration, and diabetic retinopathy. It acts by reducing the elevated VEGF levels secondary to hypoxia and severe inflammation, thereby improving tissue perfusion. (Wang et al., 2004). This might help in subsidence of pulmonary edema in COVID-19 patients. Qilu Hospital of Shandong University, China is conducting two clinical trials of bevacizumab, both of which are expected to be over by May 2020. Thus, bevacizumab holds promise as a potential therapeutic option in the treatment of severe COVID-19 patients.

##### Meplazumab

Studies till date recognize angiotensin converting enzyme 2 (ACE2) as the major entry portal for SARS-CoV-2. However, a novel route of viral invasion through direct interaction between the SARS-CoV-2 spike protein and CD147, also known as EMMPRIN, expressed on epithelial cells has been recently described by Wang K. et al. (2020) Meplazumab (Ketantin, Jiangsu Pacific Meinuoke Biopharmaceutical Co. Ltd.) is a humanized IgG2 monoclonal antibody against CD147 that has demonstrated dose-dependent inhibitory action on SARS-CoV-2 replication and virus-induced cytopathic effect *in vitro* (Bian et al., 2020). CD147 binds to cyclophilin A (CyPA), a pro-inflammatory cytokine up-regulated in viral infection, and regulates cytokine secretion and leukocyte chemotaxis. Meplazumab is a monoclonal anti-CD147 antibody that inhibits CyPA-induced T cell chemotaxis and thus reduces local inflammation. Bian et al. (2020) studied the effects of meplazumab in 17 hospitalized patients with COVID-19 at Tangdu hospital, China, and reported that meplazumab treatment significantly improved the clinical outcomes in severely ill patients. Also, the time to virus negativity in the meplazumab group was shortened compared to the control group. These evidences suggest that meplazumab therapy improves the recovery of patients with SARS-CoV-2 pneumonia and has a favorable safety profile. The drug is currently in phase II clinical trial, which is expected to be completed by December 2020.

##### Itolizumab

Itolizumab (Alzumab, Biocon Ltd.) is a humanized anti-CD6 IgG1 monoclonal antibody that was introduced in India in 2013 for the treatment of chronic plaque psoriasis. It binds specifically to domain 1 of CD6 and modulates the activation and proliferation of T cells by CD6 co-stimulation, without interfering with the interaction between CD6 and activated leukocyte-cell adhesion molecule. It inhibits intracellular phosphoproteins like mitogen-activated protein kinase (MAPK) and STAT3 and interferes with CD6-mediated intracellular signaling pathways and Th17 development. Itolizumab downregulates the transcription of pro-inflammatory cytokine genes and thus leads to decreased levels of IFN-γ, IL-6, and TNF-α, causing attenuation of cytokine storm and T cell infiltration (Menon and David, 2015). Considering its unique mechanism of action, the drug has been repurposed for the treatment of CRS, which is the leading cause of death in COVID-19. A prospective, multi-centric, randomized phase II study conducted on 30 severely ill COVID-19 patients (20 cases and 10 controls) in India showed significant improvement in blood oxygen levels with reduced levels of proinflammatory cytokines and reduced mortality rate in patients who received itolizumab. A similar trial conducted in Cuba also indicated positive results with 79.2% of the patients discharged from ICU after 2 weeks of treatment.^10^ Itolizumab has been approved by Drugs Controller General of India for the treatment of CRS in moderate to severe ARDS patients with COVID-19.

#### Anakinra

Anakinra (Kineret; Amgen Inc.) is a recombinant human IL-1 receptor antagonist that competitively inhibits the binding of IL-1α and IL-1β to the high-affinity IL-1 receptor. It is the first biological agent approved for the treatment of rheumatoid arthritis. It is administered through subcutaneous route and has an absolute bioavailability of 95% (Cvetkovic and Keating, 2002). In COVID-19 patients, halting the disease progression from manageable hypoxia to frank respiratory failure and ARDS can have a significant impact on patient management and outcomes. Therefore, a therapy directed at intercepting the cytokine storm may be beneficial in this regard. There is an ongoing prospective, randomized, interventional trial comparing the therapeutic effects of individual and simultaneous blockade of IL-6 and IL-1 versus standard care in COVID-19 patients. The trial will include 342 participants whose clinical status after 15 days of treatment will be assessed to measure the effectiveness of anakinra alone and in combination with tocilizumab and siltuximab in restoring lung homeostasis.^11^ The study is estimated to be completed in December 2020. Considering the role of IL-1 in the pathogenesis of acute lung injury in COVID-19, anakinra seems to be a promising therapeutic option in the management of such patients.

#### Cellular Therapies

##### Mesenchymal Stem Cells

Several studies have recognized the potential benefits of cell-based therapies in a number of disease processes including pulmonary, cardiovascular, hepatic, renal, metabolic, and mulculoskeletal disorders. A guideline published by the Italian College of Anesthesia, Analgesia, Resuscitation and Intensive Care has mentioned that stem cells have the potential to decrease ICU admission and curtail the number of ICU days in COVID-19 (Vergano et al., 2020). Currently, USFDA recommends autologous bone marrow stem cells as the only candidate for stem cell therapy. Mesenchymal stem cells (MSCs) have shown benefit in the treatment of musculoskeletal disorders such as low-back pain and spinal injuries. The other stem cells that can be considered for clinical use include adipose, amniotic, and umbilical cord stem cells. Among these, umbilical cord stem cells seem to be the more attractive as unlike bone marrow, umbilical cord (Wharton jelly) has a high concentration of MSCs that can be extracted noninvasively (Arutyunyan et al., 2016). Moreover, they have fast doubling times, more plasticity, greater potency, and can be efficiently be expanded in the laboratory to cater the large number of expected coronavirus patients (Nagamura-Inoue and He, 2014). Despite being allogenic, MSCs can evade the host immune system as they express low levels of MHC I, MHC II, and T cell co-stimulatory molecules, CD80 and CD86, on their surface. At a cellular level, MSCs demonstrate powerful immunomodulatory activity through secretion of anti-inflammatory molecules by paracrine effect and direct interaction with T and B lymphocytes, dendritic cells, macrophages, and NK cells. All these may help in attenuating the cytokine storm (Tipnis et al., 2010). They suppress the hyperactive immune system and promote endogenous repair by improving the cellular microenvironment. Multiple studies have demonstrated the beneficial effects of MSCs in the settings of ALI and ARDS. When given intravenously, MSCs accumulate in the lungs and improve lung function by decreasing inflammation, reducing pulmonary endothelial permeability, facilitating alveolar fluid transport, preventing pulmonary fibrosis, and promoting tissue repair. Several clinical trials have documented the safety and efficacy of MSCs in immune-mediated inflammatory diseases, such as graft versus-host disease (GVHD) and autoimmune disorders (Li et al., 2016; Atluri et al., 2020; Behnke et al., 2020). MSCs secrete antimicrobial peptides and proteins (AMPs) such as cathelicidin LL-37, human beta-defensin-2 (hBD-2), hepcidin, and lipocalin-2 (Lcn2) and anti-inflammatory molecules such as indoleamine 2,3-dioxygenase (IDO) and interleukin (IL)-17. AMPs cause disruption of membrane integrity, inhibition of protein and nucleic acid synthesis, and blockade of interaction with intracellular targets (Alcayaga-Miranda et al., 2017). MSCs regulate the host immune response by maintaining a dynamic equilibrium between pro- and anti-inflammatory cytokines. There was a concern that SARS-CoV-2 can infect the stem cells and render them ineffective. However, a study of seven COVID-19 patients (one critically ill, four serious and two mild) in Beijing revealed that SARS-CoV-2 was not able to infect the injected umbilical cord MSCs. All patients who received single dose of stem cell therapy recovered during the 14 days follow-up period, while two out of three patients (with serious disease) who did not receive stem cell therapy (control group) had unfavorable outcomes (one died and one developed ARDS). There was gradual normalization of oxygen saturation and levels of inflammatory biomarkers like CRP, aspartic aminotransferase, creatine kinase, and myoglobin in the treated group with no treatment-related adverse events. Follow-up CT scan of lungs showed significant radiological improvement (Leng et al., 2020). Thus, MSCs can be a safe and effective treatment option for patients with COVID-19 pneumonia.

#### Natural Killer Cells

Natural killer (NK) cells (large granular lymphocytes) are innate lymphocyte subsets that constitute the frontline defence system against virus infected and tumor cells. They originate in the bone marrow and represent up to 15% of peripheral blood mononuclear cells. NK cells are phenotypically defined by expression of CD56 and absence of CD3 and do not require prior stimulation to perform their effector functions. NK cells display a diverse range of biological activities that are controlled by several inhibitory and activating receptors. The inhibitory receptors recognize self-MHC class I and prevent NK cell activation. In viral infections, there is upregulation of activating receptors and downregulation of MHC class I expression, which causes activation of NK cells. The major activating receptors include cytotoxicity receptors (NKp46 and NKp44), C-type lectin receptors, and immunoglobulin-like receptors. Among the inhibitory receptors, the killer immunoglobulin-like receptors and leukocyte inhibitory receptors have prominent role in defence against viral infections. NK cells lack antigen-specific receptors and kill virus-infected cells through the production of cytokines (TNF-α, GM-CSF, CCL5/RANTES, and IFN-γ), perforin-granzyme-mediated cellular destruction, and death receptor-mediated cytolysis (Cooper et al., 2001). Perforin, a pore forming protein, increases the cell permeability, which allows granzymes, a family of serine proteases, to enter into the cell and disrupt cell cycle progression, inflict DNA damage, and promote karyolysis (Vivier et al., 2008). They also cause recruitment and activation of other effector cells, including CD8+ T cells and CD4+ Th 1 cells. Patients with deficient NK cell response are predisposed to recurrent viral infections (Jost and Altfeld, 2013). Currently, the role of NK cells for immunotherapy in infectious diseases is being explored and results seem to be promising. As hunt for new therapeutic options in the treatment of COVID-19 continue to expand, focus has been on the potential benefits of NK cell-based therapy. On April 3, 2020, USFDA approved the use of CYNK-001, the only cryo-preserved allogeneic NK cell therapy, derived from placental hematopoietic stem cells, in adults with COVID-19. The agent’s manufacturer Celularity, a New Jersey–based therapeutic company, in collaboration with Sorrento Therapeutics is about to launch a phase I/II clinical trial on CYNK-001, involving 86 COVID-19 patients.^12^ The therapy is already being tested in patients with acute myeloid leukemia, multiple myeloma, and various solid tumors. In January 2020, Celularity’s CYNK-001 was approved by USFDA for treatment of glioblastoma multiforme. Thus, considering the potent antiviral and immunomodulatory properties of NK cells, their efficacy in the treatment of COVID-19 seems promising and needs to be evaluated in clinical trials.

### Convalescent Plasma Therapy

Convalescent plasma therapy (CPT) is a passive immunization strategy that has been used for the prevention and treatment of several infectious diseases for more than a century. CPT has been successfully used in the treatment of SARS (Cheng et al., 2005), MERS (Ko et al., 2018), and influenza A H1N1 (Hung et al., 2011), with satisfactory efficacy and safety profile. A protocol for the use of convalescent plasma (CP) in the treatment of MERS was established in 2015. CPT is associated with a significant reduction in viral load and pooled mortality as revealed in a large meta-analysis on SARS and severe influenza (Mair-Jenkins et al., 2015). In 2014, WHO recommended the use of CP as an empirical treatment for Ebola virus disease during outbreaks.^13^ However, CPT did not offer much survival benefit in Ebola virus disease, as data on neutralizing antibody (NAb) titers were not available for stratified analysis. Since SARS-CoV-2 shares virological and clinical similarities with SARS-CoV and MERS-CoV, and NAbs play a crucial role in virus clearance, CPT might hold promise in the treatment of critically ill COVID-19 patients. Patients with a high titer of NAb, after having recovered from COVID-19 may be a valuable donor for CP. It has been observed that the NAbs titers in COVID-19 patients remain low for the first 10 days following disease-onset and tends to increase thereafter, reaching a peak in 12 to 15 days after the onset (Wu et al., 2020). USFDA has laid down eligibility criteria for COVID-19 CP donors which include: i) evidence of confirmed COVID-19 documented by a positive nasopharyngeal PCR at the time of illness or a positive SARS-CoV-2 antibody test after recovery, ii) complete resolution of symptoms at least 28 days prior to donation or at least 14 days prior to donation and negative results for COVID-19, either from a nasopharyngeal swab specimen or by a molecular diagnostic test from blood, iii) Male/female donors tested negative for HLA antibodies, and iv) SARS-CoV-2 neutralizing antibody titers of ≥1:160.^14^ In a study from China, CPT supplemented with supportive care and antiviral agents was associated with significant clinical and radiological improvement with a rise in neutralizing antibody titers and a fall in C-reactive protein levels within 7 days of initiation of treatment. No treatment-related adverse effects were observed (Duan et al., 2020). Similar findings were reported by Shen et al. (2020). A systematic review on CPT for the treatment of COVID-19 revealed that CPT is safe, effective, and reduces mortality in critically ill patients (Rajendran et al., 2020). A clinical trial evaluating the benefits of CP in the treatment of COVID-19 is being conducted by Universidad del Rosario, Colombia (NCT04332380), the results of which are expected to be declared by December 2020.

### CytoSorb Therapy

CytoSorb (CytoSorbents Corp.) is an extracorporeal cytokine adsorber that acts by removing the circulating cytokines and redirecting the activated neutrophils to the site of infection. This may help in ameliorating cytokine storm that can otherwise trigger uncontrolled systemic inflammatory response, organ failure, and death. CytoSorb offers significant survival benefits in septic shock as observed in several studies. It has been safely used in over 80,000 cases worldwide, primarily in the treatment of several immune-mediated life-threatening conditions such as septic shock, influenza, ARDS, secondary HLH, liver failure, and pancreatitis. CytoSorb helps in protecting endothelial tight junctions, thus reducing capillary leak syndrome. It also modulates pulmonary metabolism, edema formation, and cell-mediated infiltration and injury to the lungs.^15^ On April 10, 2020, the USFDA approved emergency use of CytoSorb for the treatment of adult COVID-19 patients admitted to ICU with features of respiratory failure.^16^ SARS-CoV-2 can induce a sepsis-like syndrome, and in such cases, since pharmacological approaches fail to give promising results, removal of pro-inflammatory cytokines by hemoadsorption through CytoSorb should be considered. To date, more than 200 critically ill patients with COVID-19 infection have been treated with CytoSorb across various centers in Italy, China, and Germany. Based on positive results in Italy, the Brescia Renal COVID Task Force has formally recommended the use of CytoSorb in severe COVID-19 patients with stage 3 acute kidney injury, receiving continuous renal replacement therapy (CRRT). CytoSorb therapy has also been recommended by the National Guidelines for the Care of Adult Patients COVID-19, Panama. In addition, the Handbook of COVID-19 Prevention and Treatment, issued by Zhejiang University School of Medicine, China is also recommending CytoSorb therapy for the management of cytokine storm in critically ill COVID-19 patients.^15^ Currently, an ongoing clinical trial (NCT04324528) is investigating the efficacy of CytoSorb in the treatment of patients with severe COVID-19 disease.^17^ It is expected to be completed by November 2020.

## Conclusion

Formulating appropriate treatment strategies for COVID-19 poses a considerable challenge. During pandemics, in absence of clinically proven treatment guidelines, the tendency is to repurpose drugs based on their antiviral and immunomodulatory activities, as evidenced through observational studies. However, such studies have certain drawbacks like lack of concurrent controls, ill-defined patient selection criteria, small sample size without randomization, and use of intermediary outcomes like viral clearance rather than patient-relevant outcomes. Though several repurposed drugs have shown promising results, and their potential clinical benefits appear to outweigh the relatively minor risk of adverse events, conclusive evidence is lacking. There is a need to clearly define the patient populations who warrant therapy and the timing of initiation of treatment. Since viral loads are highest early in the course of infection and the disease progression can occur rapidly in stable patients, it is rational to consider rapid initiation of therapy in high-risk populations (old age, hospitalized patients, those with underlying diseases and comorbidities), ideally in the context of a well-controlled, randomized clinical trial. Moreover, the demand for unproven therapies can cause shortages of medications that are otherwise indicated for more prevalent diseases like HIV, malaria, hypertension, and diabetes mellitus. The IDSA guidelines for treatment of patients with COVID-19 raise concern upon these aspects. In an attempt to generate and disseminate clinical data on an urgent basis, a phenomenal increase in fast-track publications related to COVID-19 has been observed. However, caution should be exercised because the bulk of the available clinical data are often uncontrolled, not peer reviewed, and subject to publication bias (with an intention to publish outstanding results, there may be a tendency to publish positive outcomes and disregard the negative findings). There are several ongoing clinical trials, some with versatile designs that can reasonably explain the therapeutic benefits offered by these drugs in the management of COVID-19. Given the plethora of uncertainties concerning the reliability of existing data and the safety and efficacy of the proposed treatments, it would be wise to wait for the results of clinical trials than to adopt clinically unproven therapies.

## Author Contributions

Conceptualization, data curation, and methodology: AS, MG, and VN. Supervision: AS and VN. Validation and visualization: AS, MG, and VN. Writing (original draft): AS and MG. Writing (review) and editing: AS, MG, and VN.

## Conflict of Interest

The authors declare that the research was conducted in the absence of any commercial or financial relationships that could be construed as a potential conflict of interest.

## Footnotes

1 World Health Organization (WHO). Coronavirus disease (COVID-19): WHO Director-General’s opening remarks at the media briefing on COVID-19 - 28 February 2020. Geneva: WHO; 28 February 2020. Available from: https://www.who.int/dg/speeches/detail/who-director-general-s-opening-remarks-at-the-media-briefing-on-covid-19---28-february-2020 (Accessed August 18, 2020).

2 World Health Organization (WHO). Coronavirus disease (COVID-19): Events as they happen. Geneva: WHO; 11 March 2020. Available from: https://www.who.int/emergencies/diseases/novel-coronavirus-2019/events-as-they-happen (Accessed August 18, 2020).

3 World Health Organization (WHO). Coronavirus disease (COVID-19): situation report, 209. Geneva: WHO; 18 August 2020. Available from: https://www.who.int/emergencies/diseases/novel-coronavirus-2019/situation-reports/ (Accessed August 18, 2020).

4 European Centre for Disease Prevention and Control. Novel coronavirus disease 2019 (COVID-19) pandemic: increased transmission in the EU/EEA and the UK – sixth update – 12 March 2020. Stockholm: ECDC; 2020. Available from: https://www.ecdc.europa.eu/sites/default/files/documents/RRA-sixth-update-Outbreak-of-novel-coronavirus-disease-2019-COVID-19.pdf (Accessed August 18, 2020).

5 World Health Organization (WHO). Coronavirus disease (COVID-19): Kluge, H.H.P. Statement – Older people are at highest risk from COVID‐19, but all must act to prevent community spread. Geneva: WHO; 02 April, 2020. Available from: http://www.euro.who.int/en/health‐topics/health‐emergencies/coronavirus‐covid‐19/statements/statement‐older‐people‐are‐at‐highest‐risk‐from‐covid‐19,‐but‐all‐must‐act‐to‐prevent‐community‐spread (Accessed August 18, 2020).

6 ClinicalTrials.gov [Internet] Identifier NCT04260594. Bethesda (MD): National Library of Medicine (US); 2020 Mar 12. Clinical Study of Arbidol Hydrochloride Tablets in the Treatment of Pneumonia Caused by Novel Coronavirus. Available from: https://clinicaltrials.gov/ct2/show/NCT04260594 (Accessed July 13, 2020).

7 ClinicalTrials.gov [Internet] Identifier NCT04255017. Bethesda (MD): National Library of Medicine (US); 2020 Mar 12. A prospective, randomized controlled clinical study of antiviral therapy in the 2019-nCoV pneumonia. Available from: https://www.clinicaltrials.gov/ct2/show/NCT04255017 (Accessed July 13, 2020).

8 ClinicalTrials.gov [Internet] Identifier NCT04252885. Bethesda (MD): National Library of Medicine (US); 2020 Mar 12. The efficacy of lopinavir plus ritonavir and arbidol against novel coronavirus infection (ELACOI) Available from: https://clinicaltrials.gov/ct2/show/study/NCT04252885 (Accessed July 13, 2020).

10 ctri.nic.in [Internet] Identifier CTRI/2020/05/024959. ICMR-National Institute of Medical Statistics: Clinical Trials Registry- India; 2020 May 01. Efficacy and Safety of Itolizumab in COVID-19 Complications. Available at: http://ctri.nic.in/Clinicaltrials/showallp.php?mid1=42878&EncHid=&userName=itolizumab. Accessed July 13, 2020.

11 ClinicalTrials.gov [Internet] Identifier NCT04330638. Bethesda (MD): National Library of Medicine (US); 2020 Apr 1. Treatment of COVID-19 Patients with Anti-interleukin Drugs (COV-AID). Available from: https://clinicaltrials.gov/ct2/show/NCT04330638 (Accessed July 13, 2020).

12 Celularity Announces FDA Clearance of Landmark IND for CYNK-001, an Allogeneic, Off-the-Shelf Cyropreserved NK Cell Therapy [news release]. Warren, N.J. (2020). Published: January 22, 2020. Available at: businesswire.com/news/home/20200122005061/en/Celularity-Announces-FDA-Clearance-Landmark-IND-CYNK-001 (Accessed July 13, 2020).

13 WHO. Use of convalescent whole blood or plasma collected from patients recovered from Ebola virus disease for transfusion, as an empirical treatment during outbreaks. 2014. http://apps.who.int/iris/rest/bitstreams/604045/retrieve (Accessed July 13, 2020).

14 U.S. Department of Health and Human Services, Food and Drug Administration, Center for Biologics Evaluation and Research. Recommendations for Investigational COVID-19 Convalescent Plasma. Available at: https://www.fda.gov/vaccines-blood-biologics/investigational-new-drug-ind-or-device-exemption-ide-process-cber/recommendations-investigational-covid-19-convalescent-plasma (Accessed July 13, 2020).

15 COVID-19 and CytoSorb Therapy. Available at: https://cytosorb-therapy.com/en/covid-19/ (Accessed July 13, 2020).

16 U.S. Food and Drug Administration. FDA has authorized the emergency use of CytoSorb 300 mL device for Emergency Treatment of COVID-19. Available at: https://www.fda.gov/media/136866/download (Accessed July 13, 2020).

17 ClinicalTrials.gov [Internet] Identifier NCT04324528. Bethesda (MD): National Library of Medicine (US); 2020 Mar. 27. Cytokine Adsorption in Severe COVID-19 Pneumonia Requiring Extracorporeal Membrane Oxygenation (CYCOV). Available at: https://clinicaltrials.gov/ct2/show/NCT04324528 (Accessed July 13,2020).
